# Kynurenic Acid Is a Predictive Prognostic Metabolic Marker in ST-Elevation Myocardial Infarction

**DOI:** 10.1155/cdr/9123654

**Published:** 2025-07-17

**Authors:** Xiaolin Zhang, Miaohan Qiu, Kun Na, Minghui Cheng, Haixu Song, Ning Zhao, Dan Liu, Chenghui Yan, Yaling Han

**Affiliations:** State Key Laboratory of Frigid Zone Cardiovascular Disease, Cardiovascular Research Institute, Department of Cardiology, General Hospital of Northern Theater Command, Shenyang, China

**Keywords:** kynurenic acid, metabolic marker, NMR, prognostic, ST-acute myocardial infarction

## Abstract

**Background:** The tryptophan/kynurenic acid (KYNA) pathway plays a crucial role by modulating inflammation, oxidative stress, and immune activation. The clinical value of tryptophan metabolites in the KYNA pathway for the early diagnosis and prognosis of STEMI patients, as well as the underlying functional mechanisms, remains to be elucidated.

**Objectives:** This study evaluated the prognostic value of KYNA, a metabolite of the tryptophan pathway, in STEMI patients.

**Methods:** Untargeted metabolomics by ^1^H-nuclear magnetic resonance (NMR) analysis was used to examine metabolite changes between 50 control subjects and 50 STEMI patients with an onset time of < 3 h. Furthermore, targeted metabolomic analysis was employed to investigate the association between KYNA and the prognosis of STEMI patients by LC-Q-TOF MS analysis.

**Results:** Fifteen differential metabolites were identified between STEMI patients and control subjects by ^1^H-NMR analysis. KYNA as an important metabolite upregulated obviously in the tryptophan pathway was 337.67 nmol/L in STEMI patients (interquartile range: 241.16–500.29 nmol/L). In addition, KYNA was significantly associated with major adverse cardiovascular events (MACEs) (HR: 5.95, 95% CI: 2.03–17.44; *p* = 0.0012) and all-cause mortality (HR: 7.11, 95% CI: 1.52–33.29; *p* = 0.013) and showed moderate predictive value for 12-month MACE (area under the curve (AUC) = 0.72, 95% CI: 0.65–0.80) and all-cause mortality (AUC = 0.74, 95% CI: 0.65–0.83). KAT1 expression was upregulated in infiltrating macrophages of thrombus tissue coming from the culprit coronary artery of STEMI patients. KAT1 upregulation was also observed in macrophages located within the peri-infarct myocardium.

**Conclusions:** The KYNA level may correspond to the underlying status of acute myocardial infarction and is a promising biomarker for predicting STEMI progression.

## 1. Introduction

In recent decades, due to improvements in early diagnosis and treatment for ST-elevation myocardial infarction (STEMI), the occurrence of cardiovascular risk events has decreased [1, 2]. On the contrary, cardiovascular risk events remain elevated during the first year after STEMI [2, 3]. Therefore, timely and early interventions for cardiovascular risk events after postacute myocardial infarction (MI) are necessary [4]. However, the classic biochemical markers applied for STEMI patients were limited in their predictive capacity, thus exploration of potential biomarkers that identify high-risk individuals who would benefit from intensified risk mitigation measures [5]. STEMI recognized as a metabolically influential disease is distinguished by substantial alterations in metabolic products. Thus, exploring the novel therapeutic targets in STEMI patients has profound implications for the development of personalized medicine [5, 6]. Inflammation, oxidative stress, and immune activation contribute to the initiation and progression of STEMI [7, 8]. The tryptophan converting into kynurenine via indoleamine 2,3-dioxygenase plays an important part in the autoimmune disorder such as host defense against microbial infections [9]. Kynurenic acid (KYNA) as the major metabolite of Kyn catalyzed by kynurenine aminotransferase (KAT1) is involved in several fundamental biological processes and regarded as a key factor in driving immunotolerance and immunosuppressive reactions [10]. Interferon-*γ* released by activated inflammatory cells promoting the generation of KYNA during the catabolism of tryptophan has been proven to be involved in the pathogenesis of acute coronary syndrome (ACS) [11].

KYNA releasing a brake on IL-10 production through the inhibiting Erk1/2 phosphorylation by a cAMP-dependent pathway interfered with the macrophages inflammatory response in the occurrence of ACS. KYNA has been identified as a key regulator of immune and inflammatory responses, thereby opening up promising avenues for therapeutic intervention [12]. Previous studies indicated an intriguing positive relationship between KYNA levels and adverse cardiovascular events related to atherosclerotic plaque instability and cardiovascular mortality [12]. Therefore, our study was designed to evaluate the predictive capacity of KYNA levels in a cohort of 973 STEMI patients, with a particular focus on elucidating its significance as a prognostic indicator.

## 2. Methods

### 2.1. Study Population and Design

This study was derived from a prospective, real-world, single-center cohort of consecutive STEMI patients undergoing PCI in the General Hospital of Northern Theater Command from January 2015 to April 2017. The inclusion criteria were STEMI patients receiving PCI aged ≥ 18 years. The main exclusion criteria included KYNA data could not be obtained and co-occurrence of one or more disorders or conditions (autoimmune disorders, active inflammatory conditions, severe heart failure, suspected myocarditis or pericarditis, pathologies of the hematopoietic system, advanced renal or hepatic complications, and/or immunosuppressive therapy). Blood samples were collected upon arrival at the emergency department. Quantitative determination of plasma KYNA levels were carried out using ultraperformance liquid chromatography (UPLC)/Q-TOF analysis. During the hospitalization, therapeutic management was made by the attending physicians based on current guidelines. The study protocol (Ethics Review No. 09 [2013]) was formally approved by the Ethics Committee at the General Hospital of Northern Theater Command. This study complied with the provisions of the Declaration of Helsinki. Written informed consent was obtained from all the involved participants.

### 2.2. Metabolomics Analysis Based on ^1^H-Nuclear Magnetic Resonance (NMR)

The metabolic profiles of 50 STEMI patients and 50 control subjects were conducted by the Shanghai Metabolome Institute using ^1^H-NMR analysis [13].

### 2.3. Targeted Analysis for KYNA by LC-MS/MS

Before analysis, frozen samples were first brought to ambient temperature. Two hundred microliters of the supernatant from each sample was carefully evaporated to dryness under a gentle stream of nitrogen maintained at 35°C. The dried residues were revitalized by the addition of 50 mL of a 50:50 (v/v) acetonitrile–water mixture and then filtered through a 0.22-micron membrane to remove the potential particulate matter. For chromatographic separation, the samples were analyzed utilizing an UPLC system equipped with a high-strength silica C18 column, coupled with a Q-TOF from Waters Corporation [13].

### 2.4. Outcomes

The primary outcome was major adverse cardiovascular events (MACEs) within 12 months after the procedure, defined as a composite of all-cause mortality, recurrent MI, and heart failure. Secondary outcomes included the components of MACE within 12 months. Clinical follow-up was conducted at 3, 6, 9, and 12 months by phone calls, outpatient visits, or readmission by the research staff.

### 2.5. Thrombus Tissue From the Culprit Vessels in STEMI Patients

Thrombus tissue specimens were acquired from the culprit vessel via the thrombectomy catheters (export thrombectomy catheter; Medtronic, Minneapolis, Minnesota, United States) in STEMI patients.

### 2.6. Human Peripheral Blood Monocyte Isolation Procedure

Peripheral blood mononuclear cells (PBMCs) were extracted from STEMI patients and control subjects via density gradient centrifugation using a Ficoll-based separation solution to ensure efficient purification. Furthermore, we detected the KAT1 expression in PBMCs by western blot.

### 2.7. Immunohistochemical and Immunofluorescence Staining Analysis

Paraffin-embedded specimens were analyzed by immunofluorescence staining. The specimens were incubated with primary anti-KAT1 antibody overnight at 4°C. Staining was performed using a VECTASTAIN Elite ABC kit (ZSGB-Bio Catalog No. 9720, China) following the manufacturer's instructions.

### 2.8. Statistical Analysis

The sample size was calculated to achieve the objective of determining the relationship between identified metabolites and the risk of the MACEs at 12 months after the procedure. The sample size was determined based on the study plan, which called for the inclusion of five prediction variables (anticipated) in a Cox proportional hazards regression model for MACE at 12 months after the procedure. Given that we would include five variables in our model, we required at least 50 MACEs. Thus, we proposed a total sample size of 1000 STEMI patients. This would ensure a stable model, as we would have 10 events per variable in the multivariable analyses, based upon our anticipated 5.0% event rate in the STEMI participants. The enrolled participants were stratified into tertiles based on plasma KYNA levels. Continuous variables were presented as mean values ± standard deviation or median values along with interquartile ranges (IQRs; Q1 and Q3) and compared by analysis of variance (ANOVA) or the nonparametric Kruskal–Wallis *H* test, contingent upon the data distribution. Categorical variables were expressed as frequencies (proportions) and compared using the chi-squared test or Fisher's exact test for small sample sizes. We used the Kaplan–Meier method to estimate the rates of time-to-event occurrence; comparisons between groups were performed using the log-rank test. We used the Cox proportional hazards regression models to determine the hazard ratios (HRs) and corresponding 95% confidence intervals (CIs) and scrutinized the correlations between plasma KYNA levels and clinical outcomes. For multivariate analysis, adjusted for selected patient characteristics and medical factors include age, sex, presence of hypertension, diabetes mellitus, smoking status, prior history of MI, previous PCI, earlier stroke incidents, familial history of AMI, N-terminal pro-B-type natriuretic peptide levels, left ventricular ejection fraction, hemoglobin count, serum creatinine concentration, procedural details (e.g., transradial access and the number of treated coronary arteries), and prescribed medications including P2Y_12_ inhibitors, aspirin, angiotensin-converting enzyme inhibitors/angiotensin receptor blockers, statins, and beta-blockers. The receiver operating characteristic (ROC) curve for plasma KYNA was generated to evaluate its accuracy in predicting 12-month MACE and all-cause mortality, with the discriminatory power measured by the area under the curve (AUC). The evolving discriminatory capability of KYNA for predicting MACE was assessed using a time-dependent ROC analysis. We used a restricted cubic spline (RCS) regression model to investigate the potential nonlinear relationship between plasma KYNA and the likelihood of 12-month MACE. Statistical analyses were performed using SAS software (Version 9.4; SAS Institute, Cary, North Carolina, United States). Unless otherwise specified, a two-sided *p* < 0.05 was considered to indicate statistical significance.

## 3. Results

### 3.1. Study Population

**Figure 1:**
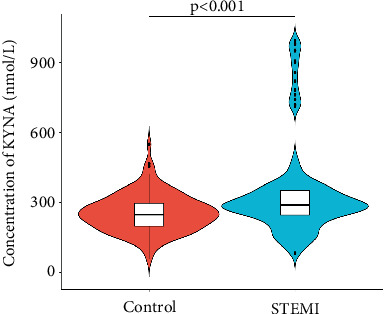
KYNA levels between the STEMI patients and control subjects.

We identified 31 key metabolites (Figure S1) in 50 STEMI patients and 50 control subjects, including sugars, amino acids, organic acids, alcohols, nucleotides, and lipids. Compared to control subjects, 15 significantly different metabolites were identified in the plasma of STEMI patients, comprising 10 amino acids, 3 organic acids, glucose-related compounds, and 1 alcohol compound. Pyroglutamic acid, 3-hydroxybutyric acid, alpha-glucose, and salicylic acid levels were elevated in STEMI patients. On the coronary, glutamic acid, tryptophan, histidine, sorbitol, formic acid, isoleucine, tyrosine, leucine, valine, glycine, and alanine levels were downregulated in STEMI patients. Among these amino acids, glutamic acid, tryptophan, and histidine had greater absolute correlation coefficients, contributing more significantly to the differences between control subjects and STEMI patients. Further study showed there were significant differences in KYNA levels between the control subjects and the STEMI patients (Figure 1).

The study included 973 STEMI patients with the median KYNA plasma concentration of 337.67 nmol/L (IQR, 241.16–500.29 nmol/L). The recruited STEMI patients' baseline characteristics, procedural information, and medication results for the KYNA tertiles were shown in Table 1. The mean age of the STEMI patients was 59.23 ± 12.31 years; males comprised 80.06%. The proportions of these patients with hypertension and diabetes were 57.30% and 31.20%, respectively. Patients with elevated KYNA levels also had higher plasma NT-proBNP levels. Medical treatment was similar among control subjects and STEMI patients (Table 1).

### 3.2. Clinical Outcomes

**Figure 2:**
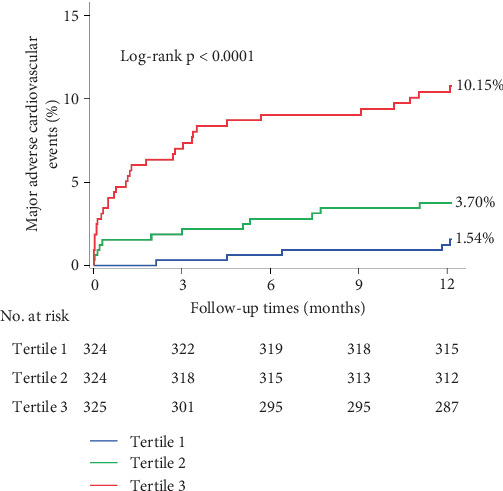
Kaplan–Meier curves for the 12-month major adverse cardiac event across tertiles of KYNA. MACE denotes a major adverse cardiac event, a composite of all-cause mortality, myocardial infarction, and heart failure.

**Figure 3:**
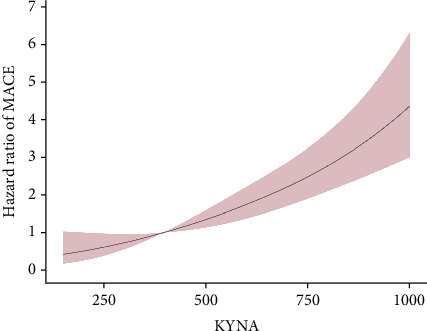
Restricted cubic spline regression analysis of KYNA for a 12-month major adverse cardiac event. HR, hazard ratio; CI, confidence interval.

Nine hundred and seventy-three STEMI patients with a 98.18% follow-up rate at 12 months were finally included in the study. The principal outcomes were presented in Table 2. The primary endpoint of 12-month MACE was observed in 5 individuals (1.54%), 12 (3.70%), and 33 (10.15%) in Tertiles 1, 2, and 3 of the KYNA group, respectively, revealing a statistically significant difference (*p* < 0.001). A significant difference was found in the frequencies of all-cause mortality (0.93% vs. 2.78% vs. 7.08%, *p* < 0.001) and heart failure (0.62% vs. 0.31% vs. 2.77%, *p* = 0.008) at 12 months among the KYNA tertiles. A Kaplan–Meier curve for the cumulative incidence of MACE at 12 months was shown in Figure 2. The plasma KYNA levels were strongly associated with increased risk of MACE (adjusted HR: 1.020; 95% CI: 1.011–1.029; *p* < 0.001) and all-cause mortality (adjusted HR: 1.023; 95% CI: 1.012–1.033; *p* < 0.001) by the multivariable analyses. Furthermore, the plasma KYNA levels were associated with MACE (highest KYNA tertile vs. lowest KYNA tertile, adjusted HR: 5.95, 95% CI: 2.03–17.44; *p* = 0.0012) and all-cause mortality (adjusted HR: 7.11, 95% CI: 1.52–33.29; *p* = 0.013) (Table 3). RCS regression analysis showed that KYNA levels were associated with 12-month MACE without a nonlinear correlation (overall *p* < 0.001; nonlinearity: *p* = 0.57) (Figure 3).

### 3.3. Predictive Performance of KYNA

**Figure 4:**
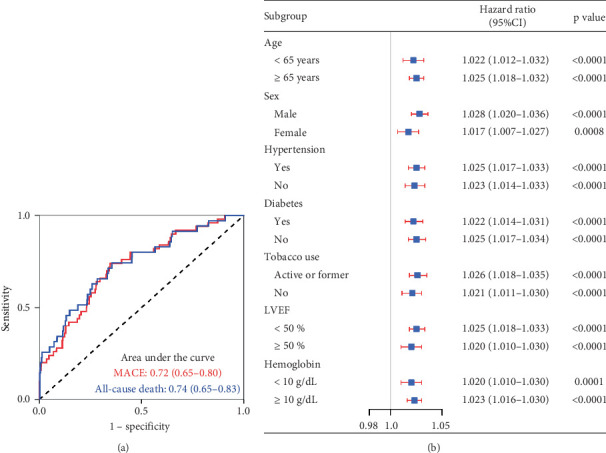
Receiver operating characteristic curves and sensitivity analysis of the associations between KYNA and major adverse cardiac events. (a) Receiver operating characteristic curves of KYNA for major adverse cardiac events and all-cause mortality. (b) Sensitivity analysis of the associations between KYNA and major adverse cardiac events.

The KYNA level showed moderate predictive value for 12-month MACE (AUC = 0.72, 95% CI: 0.65–0.80) and all-cause mortality (AUC = 0.74, 95% CI: 0.65–0.83) (Figure 4a). Time-varying ROC analysis showed that the KYNA level could robustly and reliably predict the risk of MACE within 12 months (Figure S2A). The AUCs for the 3-, 6-, 9-, and 12-month MACE were 0.79, 0.75, 0.74, and 0.72, respectively (Figure S2B). Sensitivity analyses consistently verified the association between elevated KYNA levels and the risk of MACE within a 1 year, as depicted in Figure 4b.

### 3.4. High KAT1 Expression in Coronary Artery Thrombi Tissues

**Figure 5:**
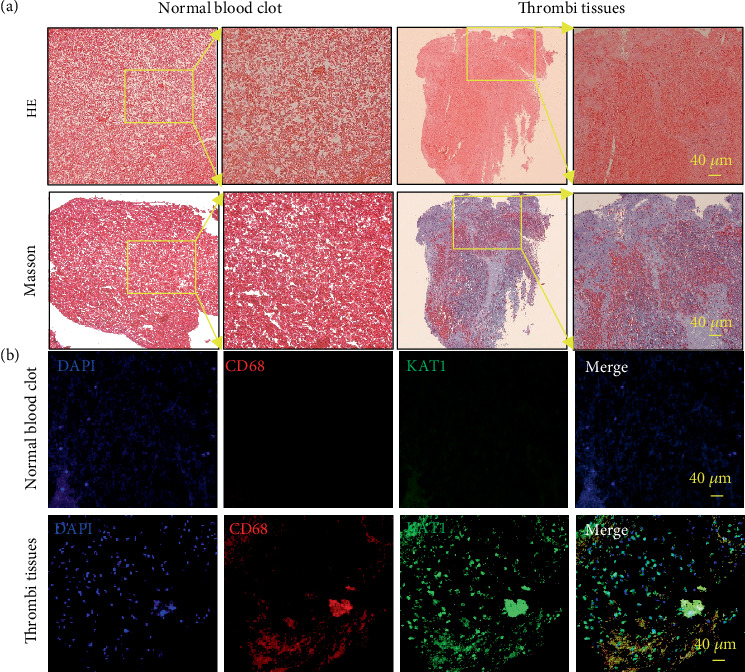
High KAT1 expression in the thrombi of coronary arteries. Coronary artery thrombi were obtained from patients with STEMI who presented to the cardiac catheterization laboratory. (a) Representative immunofluorescence staining for CD68 and KAT1 in coronary artery thrombi of patients with STEMI. Representative images show the hematoxylin and eosin (HE) and Masson's trichrome staining. (b) Merged immunofluorescence image showing CD68-positive (red) macrophages and KAT1 (green) expression. Nuclei are counterstained with DAPI. The colocalization of KAT1 and CD68-positive macrophages is depicted in the overlay. The square indicates the macrophage cells expressing KAT1.

Thrombectomy catheters were employed to extract multiple thrombotic specimens from the culprit coronary arteries, which subsequently underwent immunofluorescence staining analysis to investigate the colocalization of CD68-positive macrophages with KAT1 expression, as illustrated in Figure 5.

### 3.5. KAT1 Expression in PBMCs From STEMI Patients

**Figure 6:**
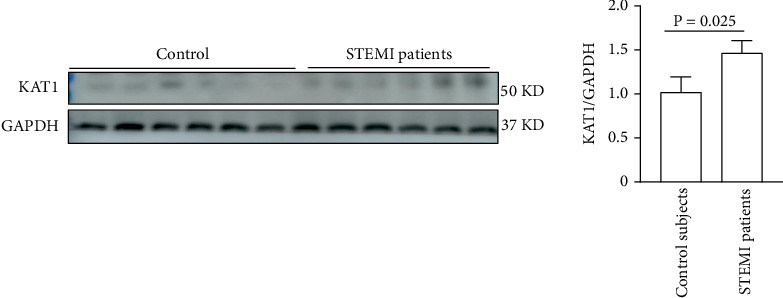
Representative western blots of KAT1 expression in PBMCs of STEMI patients and control participants. Human PBMCs were obtained from patients with STEMI and control subjects. ⁣^∗^*p* < 0.05 versus control subjects.

KAT1 expression obviously upregulated in PBMCs derived from STEMI patients was a 1.43-fold increase compared with the control subjects by western blot analysis. These findings underscored the pivotal pathological role of KAT1 in the development of STEMI, as depicted in Figure 6.

### 3.6. KAT1 Expression Increased in Cardiac Tissues After MI

**Figure 7:**
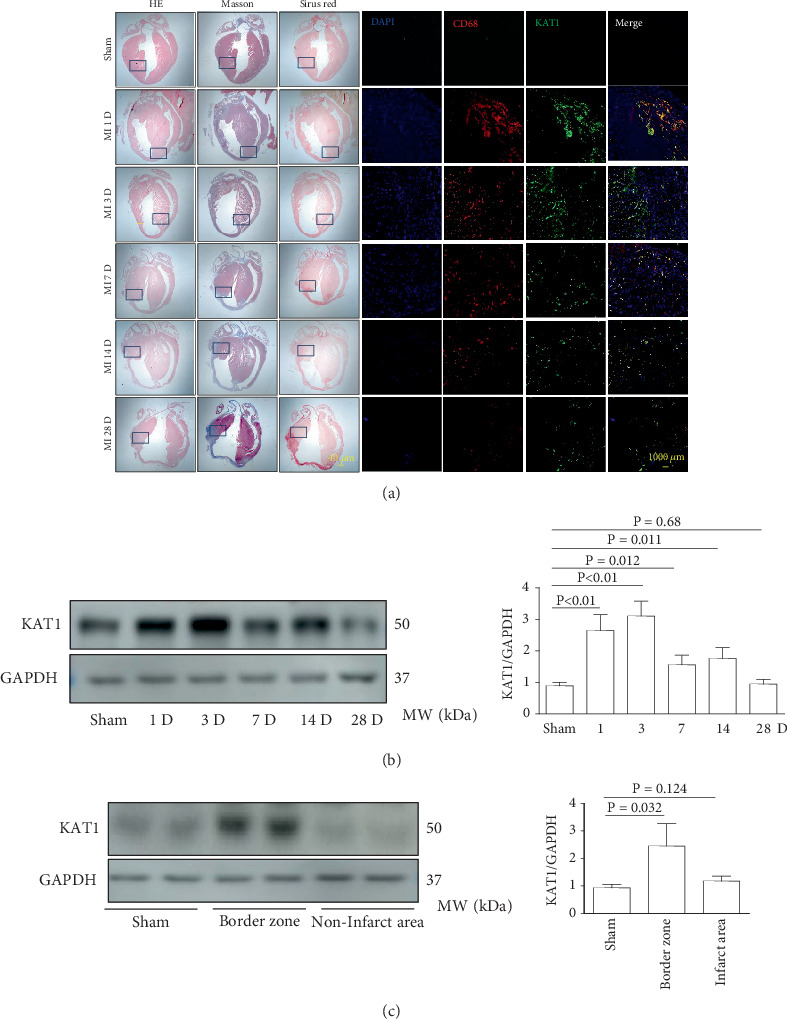
KAT1 expression in the mice cardiac tissue remodeling after MI. (a) HE, Masson's trichrome, and Sirius Red staining show that MI is induced by permanently ligating the left anterior descending coronary artery. Cardiac remodeling is assessed at 1, 3, 7, 14, and 28 days post-MI or following sham surgery without ligation. Immunofluorescence analysis reveals that KAT1 expression varies in the infarcted and noninfarcted regions at different time points after MI, with significant upregulation observed 1–3 days post-MI. (b) The KAT1 level is quantitatively assessed at various time following MI through the application of western blot analysis, *n* = 3. (c) KAT1 expression in the border zone and noninfarct areas after MI. The expression of KAT1 in both the border zone and noninfarcted regions after MI exhibited a statistically significant increase when compared to the sham group, *n* = 3.

The expression of KAT1 increased in cardiac tissues following MI. Immunofluorescence staining showed KAT1 expression in myocardial tissue at the infarct border zone of the heart tissue at different time points after MI in mice. Immunofluorescence staining showed that KAT1 expression in the infarcted area was significantly increased compared to the noninfarcted area at 1, 3, 7, 14, and 28 days after MI, especially from Days 1 to 3. Moreover, western blot analysis revealed that KAT1 expression in the infarcted area gradually increased and then decreased after 1, 3, and 7 days following MI (Figure 7).

## 4. Discussion

This study used a large real-world cohort to evaluate the association between KYNA levels and 12-month MACE in STEMI patients undergoing primary PCI.

Our study yielded several key findings. (1) The plasma KYNA levels were associated with MACE and all-cause mortality after adjustment for potential confounding factors in STEMI patients. (2) The predictive performance of KYNA was reliable and robust in evaluating the risk of MACE within 12 months. Furthermore, KAT1 catalyzing the key step of converting Kyn into KYNA was upregulated in the macrophages of thrombus tissues of the culprit vessel, suggesting that KAT1 was involved in the process of acute MI.

Metabolomics is emerging as a potent analytical tool for explaining the potential metabolic mechanism underlying the development of cardiovascular diseases and identifying predictive biomolecules linked to mortality or adverse clinical consequences following ACS events [14, 15]. KYNA levels were associated with unstable atherosclerotic plaque rupture and could predict adverse cardiovascular events in patients with acute MI. However, our study examined STEMI patients who underwent primary PCI, which had better internal consistency. Few studies had explored the predictive value of KYNA in STEMI patients and have focused on chronic kidney disease or sepsis-induced cardiac systolic dysfunction alteration.

Our study confirmed that KYNA, either as a continuous variable or when characterized by quartiles, showed the prognostic value of the MACE and all-cause mortality in STEMI patients. Furthermore, KAT1 as the rate-limiting enzyme that converted Kyn into KYNA was upregulated in macrophages of culprit artery thrombus tissues coming from STEMI patients. Furthermore, an obvious increase in KAT1 expression was observed in macrophages of the peri-infarct myocardial region, especially during the critical 1–3 days post-MI, suggesting that KAT1 fundamentally influenced the pathology underlying the occurrence of MI.

KYNA regulated the inflammatory responses of immune cell which played a pivotal role in the development of various disorders including atherosclerosis [12], Alzheimer's disease [16], Huntington's disease [17], and schizophrenia [18]. Although KYNA had been implicated in multiple disease pathways, the exact molecular mechanisms involving KYNA remained unclear. Thus, clarifying the role of KYNA in the occurrence of AMI was crucial for understanding the disease better. We found that KYNA levels were significantly increased in STEMI patients than stable coronary artery disease patients. The KYNA activation pathway was closely associated with immune system activation and inflammation response. KYNA and their common AHR receptor were enhanced in T cells and macrophages in plasma and atherosclerotic lesions. KAT1 converted kynurenine into KYNA, reducing IL-10 production during the progression of atherosclerotic plaques by activating cAMP-dependent signaling pathways and inhibiting the phosphorylation of Erk1/2. Furthermore, KYNA directly increased intracellular cAMP levels and contributed to the development of acute ischemic events. On the contrary, the role of KYNA's anti-inflammatory and antioxidant properties had also been validated, showing its dual role in reducing ischemic damage through modulation of inflammation and oxidative stress. The divergent results might be caused by the different models employed: Jung et al. used lipopolysaccharide to activate endothelial cells revealing that KYNA inhibited the inflammatory response of endothelial cells by modulating the PPAR*δ*/HO-1–dependent signaling pathway and mitigating the atherosclerotic progression [19]. Metghalchi et al. reported that KYNA limited the macrophage IL-10 production by inhibiting Erk1/2 phosphorylation and increasing intracellular cAMP in vivo to interrupt the occurrence of ACS. Thus, these findings suggested that the complexity of KYNA pathway contributed to the formation and progression of unstable plaque ruptures within atherosclerotic lesions. Our study supported KYNA's potential as a prognostic biomarker and therapeutic target, warranting future research to explore its mechanisms to enhance clinical applications. Thus, the KYNA pathway may regulate plaque destabilization, and elevated KYNA could indicate events occurring before and after STEMI. Clarifying the precise roles of KYNA in the occurrence of acute MI is essential for augmenting our insight into the mechanisms underlying the development of acute MI [20, 21].

### 4.1. Limitations

This study has several potential limitations. First, it is a post hoc analysis stemming from a single-center, prospective cohort comprising STEMI patients undergoing PCI. Despite the sample size being considered in advance and the results being adjusted using regression, there is still potential for overestimated sample size and unmeasured confounding, respectively. Second, plasma KYNA levels were measured at admission to the emergency department. Dynamic monitoring of KYNA might predict long-term risks better. Furthermore, the time-varying ROC analysis verified the KYNA levels at admission, providing a reliable and convenient tool for predicting the risk of MACE within 12 months in STEMI patients. As with all biomarkers, prospective evaluations are desirable to demonstrate their usefulness and effectiveness in clinical practice. Further functional metabolomics research, involving the discovery of KYNA through metabolite profiling and functional analysis using biological approaches, employs a bedside-to-bench strategy to translate clinical findings into laboratory studies. This approach aims to bridge the gap between identified biomarkers and their mechanistic relevance to disease pathogenesis.

## 5. Conclusion

Our analysis suggested that the tryptophan metabolite KYNA was upregulated in STEMI patients. Further investigations for KYNA will elucidate its biological mechanisms and explore its potential therapeutic implications for STEMI patients.

## Figures and Tables

**Table 1:** Baseline characteristics, procedural information, and medication results according to the tertiles of KYNA.

	**Tertile 1 (** **N** = 324**)**	**Tertile 2 (** **N** = 324**)**	**Tertile 3 (** **N** = 325**)**	**p** ** value**
Age, years	59.62 ± 12.14	59.72 ± 11.52	58.36 ± 13.20	0.65
Male	265 (81.79%)	265 (81.79%)	249 (76.62%)	0.16
Hypertension	179 (55.42%)	186 (57.41%)	192 (59.08%)	0.64
Diabetes	88 (27.33%)	106 (32.72%)	109 (33.54%)	0.18
Previous MI	23 (7.14%)	36 (11.11%)	30 (9.23%)	0.22
Previous stroke	48 (14.86%)	48 (14.81%)	58 (17.90%)	0.47
Previous PCI	8 (2.47%)	12 (3.70%)	12 (3.69%)	0.60
Smoking				0.28
Never	126 (39.13%)	123 (37.96%)	117 (36.22%)	
Active	188 (58.39%)	193 (59.57%)	204 (63.16%)	
Former	8 (2.48%)	8 (2.47%)	2 (0.62%)	
Family history of MI	9 (2.78%)	4 (1.23%)	6 (1.85%)	0.36
LVEF, %	53.78 ± 9.32	53.79 ± 9.60	53.90 ± 9.10	0.98
NT-proBNP, pg/mL	912.00 (280.55–2061.00)	951.50 (361.00–2014.00)	1191.00 (493.00–2254.00)	0.03
Hemoglobin, g/L	139.86 ± 16.83	139.79 ± 19.78	139.10 ± 17.04	0.84
Creatinine, *μ*mol/L	75.56 ± 22.79	75.79 ± 25.46	78.80 ± 30.18	0.23
Procedure information				
Transradial access	269 (83.02%)	270 (83.33%)	285 (87.69%)	0.18
Coronary arteries treated				
Left main	6 (1.85%)	6 (1.85%)	8 (2.46%)	0.82
Left anterior descending	199 (61.42%)	199 (61.42%)	213 (65.54%)	0.46
Left circumflex	77 (23.77%)	71 (21.91%)	88 (27.08%)	0.30
Right	146 (45.06%)	160 (49.38%)	140 (43.08%)	0.26
Medical treatment				
Aspirin	317 (98.45%)	321 (99.07%)	322 (99.08%)	0.68
P2Y12 inhibitors				
Clopidogrel	207 (64.29%)	216 (66.67%)	190 (58.46%)	0.08
Ticagrelor	113 (35.09%)	110 (33.95%)	135 (41.54%)	0.10
Statin	312 (96.30%)	318 (98.15%)	312 (96.00%)	0.24
ACEI/ARB	247 (76.23%)	246 (75.93%)	240 (73.85%)	0.74
*β*-Blockers	255 (78.70%)	259 (79.94%)	264 (81.23%)	0.72
KYNA(nmol/L)	215.73 (173.07–241.03)	337.54 (301.36–379.05)	615.84 (500.29–844.04)	—

*Note:* Data are *n* (%).

Abbreviations: ACEI, angiotensin; ARB, angiotensin II receptor blockers; converting, enzyme inhibitors; LVEF, left ventricular ejection fraction; MI, myocardial infarction; PCI, percutaneous coronary intervention; proBNP, N; terminal, pro; type, natriuretic peptide.

**Table 2:** Clinical outcomes at 12 months according to the tertiles of KYNA.

	**Tertile 1 (** **N** = 324**)**	**Tertile 2 (** **N** = 324**)**	**Tertile 3 (** **N** = 325**)**	**p** ** value**
MACE	5 (1.54%)	12 (3.70%)	33 (10.15%)	< 0.001
All-cause death	3 (0.93%)	9 (2.78%)	23 (7.08%)	< 0.001
Recurrent MI	0 (0.00%)	2 (0.62%)	2 (0.62%)	0.37
Heart failure	2 (0.62%)	1 (0.31%)	9 (2.77%)	0.008

*Note:* Data are *n* (%).

Abbreviations: MACE, major adverse cardiac events; MI, myocardial infarction.

**Table 3:** Cox regression analysis of the association between KYNA and endpoints.

	**Incidence (%)**	**Crude HR (95% CI)**	**p** ** value**	**Adjusted HR (95% CI)**	**p** ** value**
MACE					
Tertile 1	5 (1.54%)	Reference	—	Reference	—
Tertile 2	12 (3.70%)	2.43 (0.85–6.88)	0.096	2.51 (0.77–8.20)	0.128
Tertile 3	33 (10.15%)	6.95 (2.71–17.80)	< 0.001	5.95 (2.03–17.44)	0.0012
KYNA per 10 (nmol/L) increase	—	1.025 (1.019–1.031)	< 0.001	1.020 (1.011–1.029)	< 0.001
All-cause death					
Tertile 1	3 (0.93%)	Reference	—	Reference	—
Tertile 2	9 (2.78%)	3.03 (0.82–11.18)	0.097	2.99 (0.58–15.48)	0.193
Tertile 3	23 (7.08%)	7.97 (2.40–26.55)	< 0.001	7.11 (1.52–33.29)	0.013
KYNA per 10 (nmol/L) increase	—	1.027 (1.020–1.034)	< 0.001	1.023 (1.012–1.033)	< 0.001

*Note:* Model adjusted for age, male, hypertension, diabetes, previous MI, previous stroke, previous PCI, smoking status, family history of AMI, LVEF, NT-proBNP, hemoglobin, creatinine, procedure information (transracial access and coronary arteries treated), and medical treatment (aspirin, P2Y12 inhibitors, statin, ACEI/ARB, and *β*-blockers).

Abbreviations: CI, confidence interval; HR, hazard ratio; MACE, major adverse cardiac events.

## Data Availability

The data that support the findings of this study are available on request from the corresponding authors. The data are not publicly available due to privacy or ethical restrictions.

## Nomenclature

^1^H NMR: ^1^H-nuclear magnetic resonance
UPLC/Q-TOF: ultraperformance liquid chromatography/electrospray ionization quadruple time-of-flight mass spectrometry
STEMI: ST-acute myocardial infarction
ACS: acute coronary syndrome
AMI: acute myocardial infarction
Trp: tryptophan
Kyn: kynurenine
KYNA: kynurenic acid
PBMCs: peripheral blood mononuclear cells

## Supporting Information

Supporting Information 1 Figure S1: Typical ^1^H-NMR spectra of serum samples from control subjects and STEMI patients. The magnified 32 times vertically was in dotted boxes, and the metabolites were represented by numbers. Note: 1. leucine, 2. isoleucine, 3. valine, 4. 3-hydroxybutyric acid, 5. lactate, 6. alanine (Ala), 7. acetate, 8. glutamate, 9. citrate, 10. creatinine, 11. choline, 12. *β*-glucose, 13. *α*-glucose, 14. tyrosine (Tyr), 15. histidine, 16. phenylalanine, 17. formate, 18. threonine, 19. tryptophan, 20. glycine, 21. lysine (Lys), 22. hypoxanthine, 23. phosphocholine, 24. pyroglutamate, 25. inosine, 26. methanol, 27. edetic acid (EDTA), 28. salicylate, 29. glucitol, 30. 2-hydroxybutyric acid (2-HB), and 31. lipid.

Supporting Information 2 Figure S2: Time-varying receiver operating characteristic curve of KYNA for major adverse cardiac events. (A) AUCs and follow-up time. (B) AUCs of the 3-, 6-, 9-, and 12-month follow-up visits. AUC, area under the curve; CI, confidence interval.
