# Supplementary figures and images for: Kynurenic Acid Is a Predictive Prognostic Metabolic Marker in ST-Elevation Myocardial Infarction
Source: Cardiovasc Ther. 2025 Jul 17;2025:9123654. doi: 10.1155/cdr/9123654 (PMC12289359; doi:10.1155/cdr/9123654)

## Slide 1
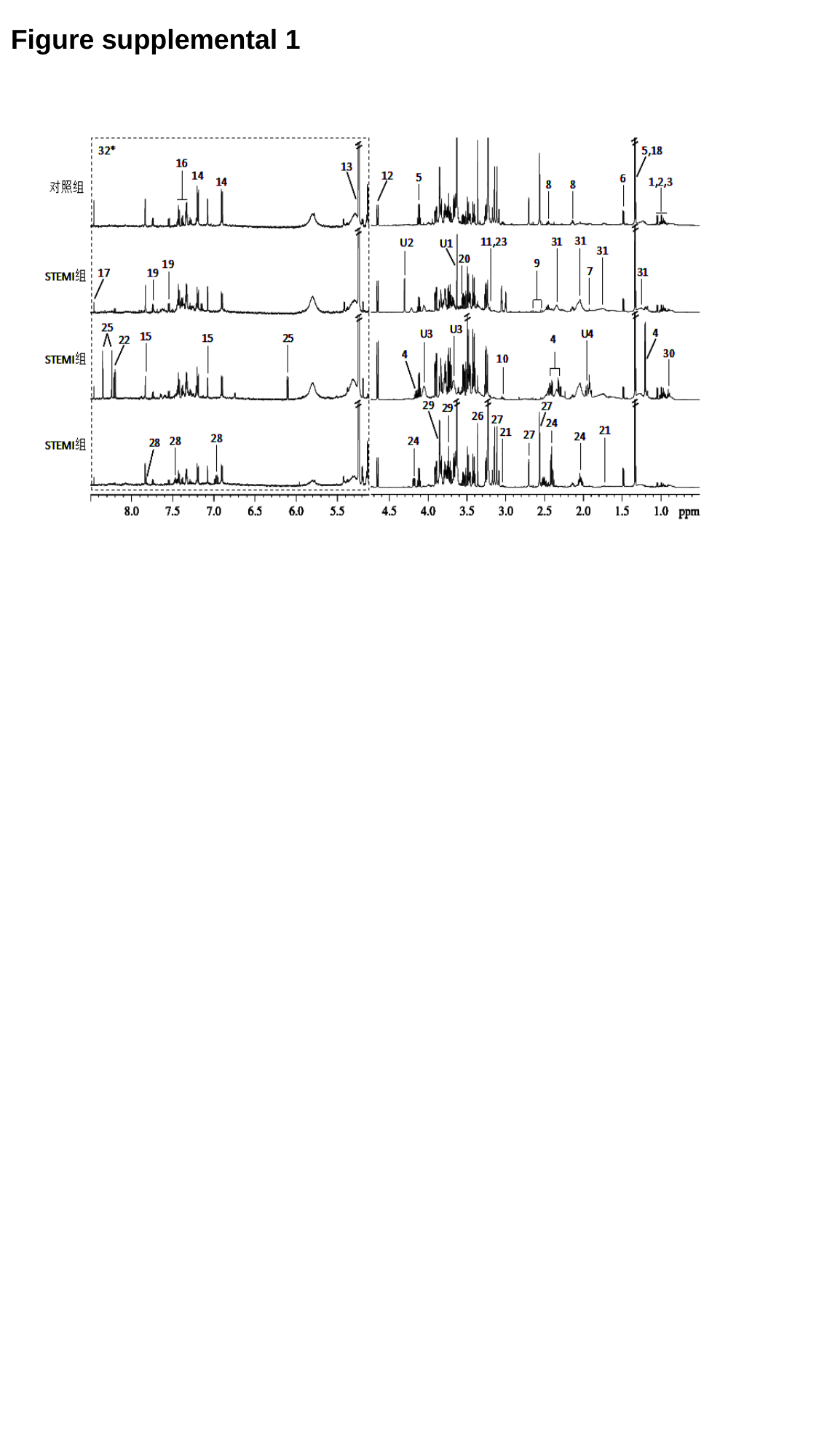

Figure supplemental 1

Supplement: Supporting Information 1 — Figure S1: Typical 1H-NMR spectra of serum samples from control subjects and STEMI patients. The magnified 32 times vertically was in dotted boxes, and the metabolites were represented by numbers. Note: 1. leucine, 2. isoleucine, 3. valine, 4. 3-hydroxybutyric acid, 5. lactate, 6. alanine (Ala), 7. acetate, 8. glutamate, 9. citrate, 10. creatinine, 11. choline, 12. β-glucose, 13. α-glucose, 14. tyrosine (Tyr), 15. histidine, 16. phenylalanine, 17. formate, 18. threonine, 19. tryptophan, 20. glycine, 21. lysine (Lys), 22. hypoxanthine, 23. phosphocholine, 24. pyroglutamate, 25. inosine, 26. methanol, 27. edetic acid (EDTA), 28. salicylate, 29. glucitol, 30. 2-hydroxybutyric acid (2-HB), and 31. lipid. [file 9123654.f1.pptx]

## Slide 1
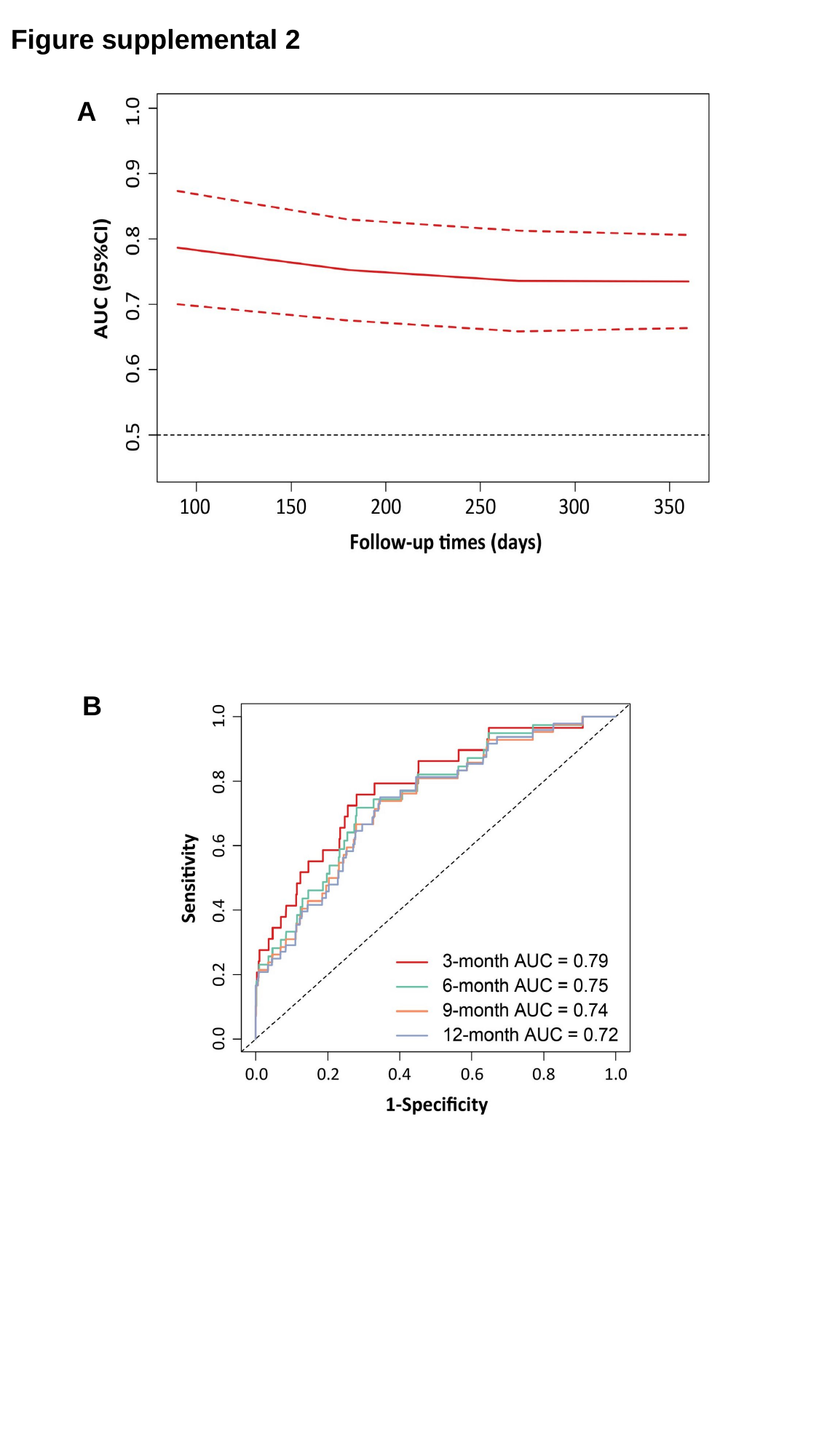

Figure supplemental 2
A
B

Supplement: Supporting Information 2 — Figure S2: Time-varying receiver operating characteristic curve of KYNA for major adverse cardiac events. (A) AUCs and follow-up time. (B) AUCs of the 3-, 6-, 9-, and 12-month follow-up visits. AUC, area under the curve; CI, confidence interval. [file 9123654.f2.pptx]
